# Genome-Wide Analysis Reveals Selection Signatures Involved in Meat Traits and Local Adaptation in Semi-Feral Maremmana Cattle

**DOI:** 10.3389/fgene.2021.675569

**Published:** 2021-04-28

**Authors:** Slim Ben-Jemaa, Gabriele Senczuk, Elena Ciani, Roberta Ciampolini, Gennaro Catillo, Mekki Boussaha, Fabio Pilla, Baldassare Portolano, Salvatore Mastrangelo

**Affiliations:** ^1^Laboratoire des Productions Animales et Fourragères, Institut National de la Recherche Agronomique de Tunisie, University of Carthage, Ariana, Tunisia; ^2^Dipartimento di Agricoltura, Ambiente e Alimenti, University of Molise, Campobasso, Italy; ^3^Dipartimento di Bioscienze, Biotecnologie e Biofarmaceutica, University of Bari “Aldo Moro”, Bari, Italy; ^4^Dipartimento di Scienze Veterinarie, University of Pisa, Pisa, Italy; ^5^Consiglio per la Ricerca in Agricoltura e l’Analisi dell’Economia Agraria (CREA), Centro di Ricerca Zootecnia e Acquacoltura, Lodi, Italy; ^6^INRAE, AgroParisTech, University of Paris Saclay, Saint Aubin, France; ^7^Dipartimento di Scienze Agrarie, Alimentari e Forestali, University of Palermo, Palermo, Italy

**Keywords:** local cattle breeds, selection signatures, diversity, candidate genes, environmental adaptation

## Abstract

The Maremmana cattle is an ancient Podolian-derived Italian breed raised in semi-wild conditions with distinctive morphological and adaptive traits. The aim of this study was to detect potential selection signatures in Maremmana using medium-density single nucleotide polymorphism array. Putative selection signatures were investigated combining three statistical approaches designed to quantify the excess of haplotype homozygosity either within (integrated haplotype score, *iHS*) or among pairs of populations (*Rsb* and *XP-EHH*), and contrasting the Maremmana with a single reference population composed of a pool of seven Podolian-derived Italian breeds. Overall, the three haplotype-based analyses revealed selection signatures distributed over 19 genomic regions. Of these, six relevant candidate regions were identified by at least two approaches. We found genomic signatures of selective sweeps spanning genes related to mitochondrial function, muscle development, growth, and meat traits (*SCIN*, *THSD7A*, *ETV1*, *UCHL1*, and *MYOD1*), which reflects the different breeding schemes between Maremmana (semi-wild conditions) and the other Podolian-derived Italian breeds (semi-extensive). We also identified several genes linked to Maremmana adaptation to the environment of the western-central part of Italy, known to be hyperendemic for malaria and other tick-borne diseases. These include several chemokine (C-C motif) ligand genes crucially involved in both innate and adaptive immune responses to intracellular parasite infections and other genes playing key roles in pulmonary disease (*HEATR9*, *MMP28*, and *ASIC2*) or strongly associated with malaria resistance/susceptibility (*AP2B1*). Our results provide a glimpse into diverse selection signatures in Maremmana cattle and can be used to enhance our understanding of the genomic basis of environmental adaptation in cattle.

## Introduction

In livestock species, domestication followed by breed formation and the subsequent artificial selection for economic and morphological traits have shaped genomic variation, driving the formation of detectable signatures on the genome of these populations (e.g., Stella et al., 2010; Qanbari et al., 2011; Rothammer et al., 2013; Signer-Hasler et al., 2017; Mastrangelo et al., 2020a). With more than 30 officially recognized local cattle breeds, Italy can be considered as one of the most important hotspots of cattle diversity (Mastrangelo et al., 2018). Several Italian breeds such as Marchigiana, Romagnola, Chianina, Calvana, Podolica, Cinisara, Modicana, and Maremmana belong to the so-called “Podolian” group (hereinafter referred to as “Podolian-derived Italian breeds”; Mastrangelo et al., 2020b). This group includes several European breeds with common phenotypic traits such as a gray coat color and long horns (Di Lorenzo et al., 2018) considered to be ancestral. Indeed, their evolutionary history seems to be linked to human Neolithic and post-Neolithic migration routes (Senczuk et al., 2020).

**Figure 1:**
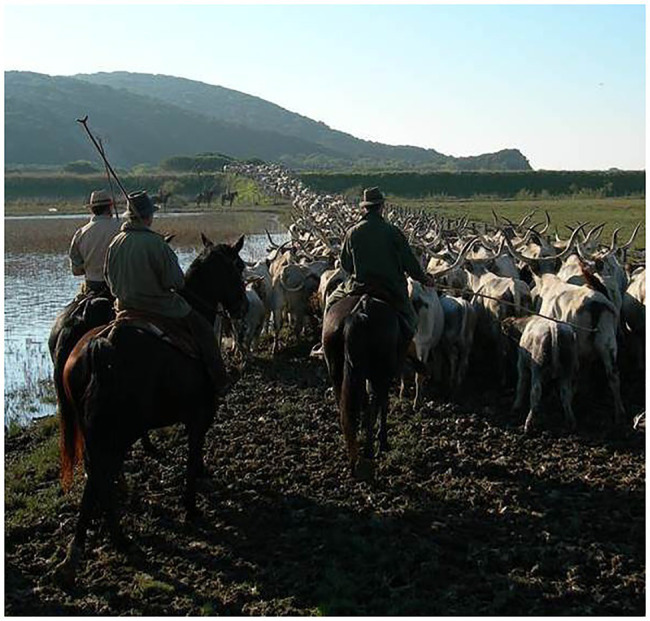
A typical herd of Maremmana cattle.

The Maremmana is an ancient cattle breed that, unlike the other Podolian-derived Italian breeds, is raised in semi-feral conditions (Figure 1), whose origin can be traced back to the Etruscan era when local would have been crossed with Podolian cattle (Maretto et al., 2012). This breed has been reared for hundreds of years in the lowlands and on the hilly areas of Maremma, a territory bordering the Tyrrhenian Sea and including much of south-western Tuscany and part of northern Lazio, in western central Italy. This region is characterized by an arid climate and poor quality pastures (Food and Agriculture Organization, 1991). The Maremma territory is also known to have been hyperendemic for malaria since the 60s of the last century (Romi et al., 2012). Until the 20th century, Maremmana cattle ran semi-wild driven by mounted herdsmen called “Vaccari” or “Butteri” (Ritchie, 2009). This breed is well-known to be resistant to several diseases such as tuberculosis, parasitic diseases (e.g., piroplasmosis), and foot and skin diseases (Food and Agriculture Organization, 1991). The Maremmana cattle, compared with other Podolian-derived Italian breeds, still display many ancestral features such sexual dimorphism, good maternal qualities, disease resistance, and very long and distinctively lyre-shaped horns. The breed has also a robust appearance characterized by a massive skeletal structure, solid legs, and hard hooves, longevity and adaptation to harsh conditions and poor-quality feed. Because of mechanization in agriculture and drainage of the marshy area, the population size of Maremmana has fallen from ~288,000, in 1944 (Consiglio Nazionale delle Ricerche, 1983) to roughly 44,000 in 1983. Since 70s, the breed was used as “maternal line” in crossbreeding programs with local and cosmopolite beef breeds. The indiscriminate and unregulated adoption of crossbreeding induced a constant and progressive depletion of pure animals to few 1,000 females (Fioretti et al., 2020). By the end of the 1980’s, this breed was nearly extinct (Food and Agriculture Organization, 2002). Later on, following the implementation of agri-environmental measures and the introduction of incentives to protect endangered livestock populations, the trend started to reverse and the number of individuals belonging to Maremmana population increased (Catillo et al., 2018). In fact, about 11,800 animals are recorded in the herdbook in 2019, including 6,853 cows and 213 bulls reared in 250 herds for beef production.^1^

Previous studies have investigated genetic diversity and population structure of Maremmana and its relationship with other breeds (e.g., Ajmone-Marsan et al., 2002; Moioli et al., 2004; Pariset et al., 2010; Maretto et al., 2012; Catillo et al., 2018; Biscarini et al., 2020; Senczuk et al., 2020), but to the best of our knowledge, there is no information on trait-specific selection signatures for this breed. Such information may contribute to improve our understanding of how selection affects livestock phenotypes, thus allowing important insights into the evolutionary history and the genetic architecture of the selected traits.

Several studies aiming at identifying genomic regions putatively under selection in cattle have been conducted by contrasting breeds with different production aptitudes (dairy and meat; e.g., Stella et al., 2010; Bomba et al., 2015; Zhao et al., 2015). Other studies have shown that it is possible to identify selection signatures by comparing breeds with similar selection goals (Sorbolini et al., 2015). The rationale behind this approach is that even though such breeds share selection goals, every breed has, in the course of time, been subjected to specific selection pressures, which might affect different set of genes.

The aim of this study was to map selection signatures in Maremmana cattle that may be linked to peculiar phenotypic characteristics. For this purpose, three statistical approaches designed to quantify the excess of haplotype homozygosity (relative to neutral expectations) were applied: (a) integrated haplotype score (*iHS*); (b) *Rsb* [standardized log-ratio of the integrated site-specific Extended Haplotype Homozygosity between pairs of populations]; and (c) *XP-EHH* (cross-population EHH). For *Rsb* and *XP-EHH* computation, the excess of long haplotypes quantified within Maremmana was contrasted with that estimated from seven other Podolian-derived Italian breeds (considered as the meta-population). *iHS* (Voight et al., 2006) detects partial selective sweeps, while *Rsb* (Tang et al., 2007) and *XP-EHH* (Sabeti et al., 2007) detect selected alleles that have risen (in frequency) to near fixation.

## Materials and Methods

### Genotyping Data, Quality Control, and Relatedness Filtering

Genotypes at loci in the Bovine SNP50K v2 BeadChip (Illumina Inc., San Diego, CA, United States) for 146 Maremmana (135 females and 11 males) and 174 pooled samples from seven other Podolian-derived Italian breeds (Marchigiana, Romagnola, Chianina, Calvana, Podolica, Cinisara and Modicana) were retrieved from previously published studies (Mastrangelo et al., 2018; Biscarini et al., 2020; Senczuk et al., 2020). In general, methods for the detection of selection signatures are designed to analyze non-related animals (Zhao et al., 2015). For this purpose, a relatedness test between individuals within each population was first performed using PLINK (Purcell et al., 2007). The software calculates a variable called PIHAT reflecting extended haplotypes shared between distantly related individuals. One individual from any pairs showing a PIHAT score ≥0.15 was removed from further analysis. After relatedness filtering, 71 Maremmana and 163 Italian Podolian-derived individuals were retained for analyses. Further quality control (QC) filtering steps to minimize the number of false-positive calls were applied. These filters included the removal of markers with minor allele frequency (MAF) ≤ 0.01 and a call rate ≤ 0.95. A total of 34,973 single nucleotide polymorphisms (SNPs) were kept after these QC steps. Moreover, samples with more than 5% of missing genotypes were also discarded from further analyses.

Because of uncertainty in the identification of the ancestral allelic state (see the section “Identification of selection signatures” below) of 3,223 SNPs in *iHS* computation, we applied this statistic to a second dataset with 31,750 SNPs. The two datasets were then subjected to linkage disequilibrium (LD) pruning using the default parameters of PLINK (SNP window size: 50, step 5 SNPs, r^2^0.5) resulting in the removal of 9,517 and 3,265 SNPs from the *iHS* and the *Rsb*/*XP-EHH* datasets, respectively. Overall, 22,233, 31,708 and 31,708 markers were used for *iHS*, *Rsb*, and *XP-EHH* computations, respectively.

### Genetic Relationships

To investigate the existence of population substructure for the breeds of the study, identity-by-state (IBS) alleles shared by all pairs of individuals were calculated using PLINK (Purcell et al., 2007) and graphically represented by multidimensional scaling (MDS).

Moreover, we computed a matrix of distances between all pairs of individuals using the ape R package (Paradis et al., 2004). The genetic distance between two individuals was defined as the number of SNPs for which they differ. A neighbor-joining tree was then computed based on the resulting distance matrix using the phyclust R package (Chen, 2011). Both analyses were carried out using the dataset containing 31,708 SNPs and 234 individuals (71 Maremmana and 163 Italian Podolian-derived).

### Identification of Selection Signatures

The three EHH-based metrics were computed using the *rehh* package in R (Gautier and Vitalis, 2012). Haplotypes were reconstructed from the genotyped SNPs using fastPHASE (Scheet and Stephens, 2006). For each chromosome, the following options were used: -T10 -Ku60 -Kl10 -Ki10, where: -T is the number of random starts of the expectation-maximization (EM) algorithm used by fastPHASE; and -Ku is the upper limit for the number of considered clusters; -Kl is the lower limit for the number of considered clusters; -Ki is the interval between values for number of clusters.

The *iHS* statistic measures the amount of EHH for a given SNP along the ancestral allele relative to the derived allele. In this study, for each SNP, the ancestral allele was inferred as the most common allele within three outgroup species including yak, buffalo, and sheep. For each SNP, an *iHS* score was computed following Voight et al. (2006). SNP scores were further transformed into two-sided *p*-values: piHS = −log10[1-2|*Φ*(*iHS*)-0.5|], where Φ (x) represents the Gaussian cumulative distribution function (assuming that the *iHS* values are normally distributed, which is expected under neutrality). We used −log10(*p*-value) = 2.5 as a threshold to define significant *iHS* values. Because it has been demonstrated that it is more powerful to look for windows of consecutive SNPs that contain numerous extreme *iHS* scores (Voight et al., 2006), *iHS* candidate regions were defined as those containing at least three neighboring SNPs exceeding the aforementioned threshold within 2-Mb sliding windows, overlapping by 20 Kb.

The *Rsb* and *XP-EHH* scores were computed between Maremmana and the other Podolian-derived breeds following Sabeti et al. (2007) and Tang et al. (2007), respectively. As in *iHS*, assuming that *Rsb* and *XP-EHH* values are normally distributed, SNP scores were further transformed into two-sided *p*-values: p*Rsb* = −log10[1–2|Φ(*Rsb*)-0.5|] and p*XP-EHH* = −log10[1-2|Φ(*XP-EHH*)-0.5|]. *Rsb* and *XP-EHH* candidate regions were defined as those containing at least four neighboring SNPs exceeding the −log10(*p*-value) = 2.5 threshold within 1-Mb sliding windows, overlapping by 10 Kb.

### Functional Characterization of Regions Identified as Under Selection

Genomic regions detected by at least two EHH-based methods were interrogated for genes annotated to the *Bos taurus* genome assembly ASR-UCD1.2 using Genome Data Viewer provided by NCBI. Database for Annotation, Visualization and Integrated Discovery (DAVID) software was used for functional enrichment analysis of the list of genes retrieved.^2^ We used an adjusted Benjamini-corrected *p*-value = 0.05 as a threshold to define significantly enriched Gene Ontology (GO) biological and functional processes. Moreover, to investigate the biological function and the phenotypes that are known to be affected by each annotated gene, we conducted a comprehensive literature search, including information from other species.

## Results and Discussion

The present study aimed at the detection of genomic regions that had been differentially selected between Maremmana and other Podolian-derived Italian breeds, which can provide clues to potential new targets of natural and artificial selection. For this purpose, a genomic scan for selective sweeps was performed by contrasting the extended haplotype homozygosity profiles between Maremmana and a single reference population composed of a pool of seven Podolian-derived Italian breeds (*Rsb* and *XP-EHH*). The third complementary EHH-based method, *iHS*, was applied because it is known to be sensitive to ongoing within-population recent selection (Voight et al., 2006).

### Genetic Relationships

**Figure 2:**
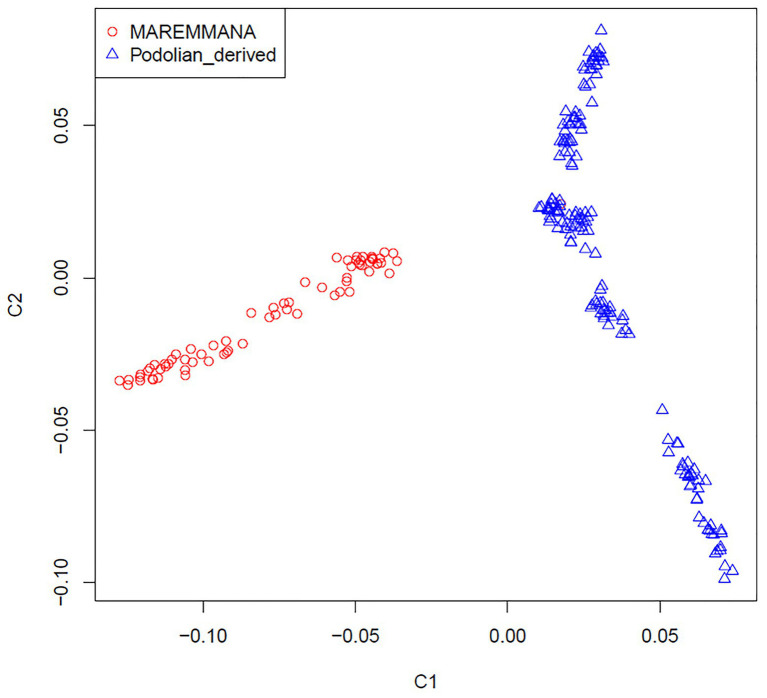
Genetic relationship defined with multidimensional scaling (MDS) analysis between Maremmana and Podolian-derived breeds.

Multidimensional scaling analysis on IBS allele-sharing values revealed a clear separation between Maremmana and the other Podolian-derived Italian breeds along the first dimension (C1; Figure 2). This clustering trend was confirmed by the neighbor joining (NJ) tree of individuals, which revealed two major clades that coincide with Maremmana and the rest of the Podolian-derived populations (Supplementary Figure S1), in agreement with previous studies (Senczuk et al., 2020; Zsolnai et al., 2020). It is also noteworthy that, both MDS and NJ tree revealed additional substructure of Maremmana suggesting the presence of individuals from several sub-populations with different allele frequencies. This genetic background is likely the result of bottleneck in the population, probably promoted by the overuse of few influential sires as parents of the next generation in the early 1990s (Porter et al., 2016), or the consequence of feral breeding. Moreover, for Maremmana breed, natural mating system is the common practice and the exchange of bulls among herds is quite unusual. This leads to an increase of inbreeding within sub-populations.

### Selection Signatures Detected by the Within-Population Test

**Figure 3:**
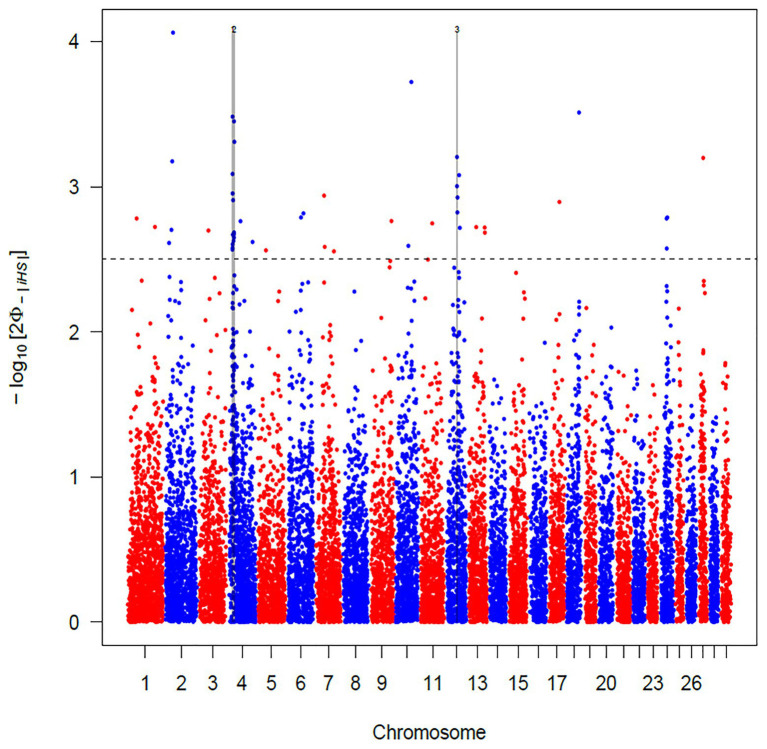
Manhattan plot of the genome-wide integrated haplotype score (*iHS*) analysis for Maremmana breed. Horizontal dashed line mark the significance threshold applied to detect the outlier single nucleotide polymorphisms (SNPs) −log10 (*p*-value) = 2.5.

The use of *iHS* test aims to identify recent and incomplete selective sweeps (Voight et al., 2006). This approach revealed a total of 103 SNPs putatively under selection in Maremmana breed. These markers defined a total of three regions, two located on BTA04 (at positions 18.44–22.80 and 24.62–28.60 Mb) and one on BTA12 (44.98–47.12 Mb; Figure 3), spanning a total of 28 and 25 known and uncharacterized genes (LOC), respectively (Supplementary Table S1). The region with the strongest signal (eight SNPs exceeding the significance threshold) is located on the BTA04 at position 18.44–22.80 Mb and spans six known genes (*THSD7A*, *TMEM106B*, *VWDE*, *SCIN*, *ARL4A*, and *ETV1*). *SCIN* and *THSD7A* genes are involved in several biological processes (BP) related to actin, an important contributor to the contractile property of muscle, such as filament capping (GO: 0051693), and severing (GO: 0051014), polymerization or depolymerization (GO:0008154) and cytoskeleton reorganization (GO:0031532). Actin is the building block of thin filaments of skeletal muscle (Lonergan et al., 2010), and together with myosin, it can affect meat quality post-mortem. A third candidate for genes under selection in the region on BTA04 is *ETV1*, a member of the PEA3 group of transcription factors, with functions related, inter alia, to muscle organ development (GO:0007517). *ETV1* is one of the strongest candidate genes for marbling score in Korean cattle (Ryu and Lee, 2016). Likewise, in a previous study analyzing the muscle transcriptome in pigs, *ETV1* was found among the top 20 differentially expressed genes between Duroc and Landrace breeds in a feed efficiency context (Carmelo and Kadarmideen, 2020).

### Selection Signatures Detected by Between-Populations Tests

The clear separation between Maremmana and the other Podolian-derived Italian breeds, in conjunction with Maremmana membership to the Podolian group suggest that between-population tests offer a powerful approach to unravel potential selection differences between them. The choice of contrasting the EHH patterns of the same haplotype between Maremmana and the seven other Podolian-derived Italian breeds relied on the distinctive primitive features of the former and its long-term adaptability to the specific environmental conditions of the Maremma plain, which houses several protozoal parasites affecting cattle (Toma et al., 2015). This region was also known to be hyper-endemic for malaria until the mid-20s (Boccolini et al., 2012).

We applied *Rsb* and *XP-EHH* tests to detect potential selective sweeps that are fixed (or nearly fixed) in Maremmana but remain segregating in the other Podolian-derived Italian breeds or vice versa.

**Figure 4:**
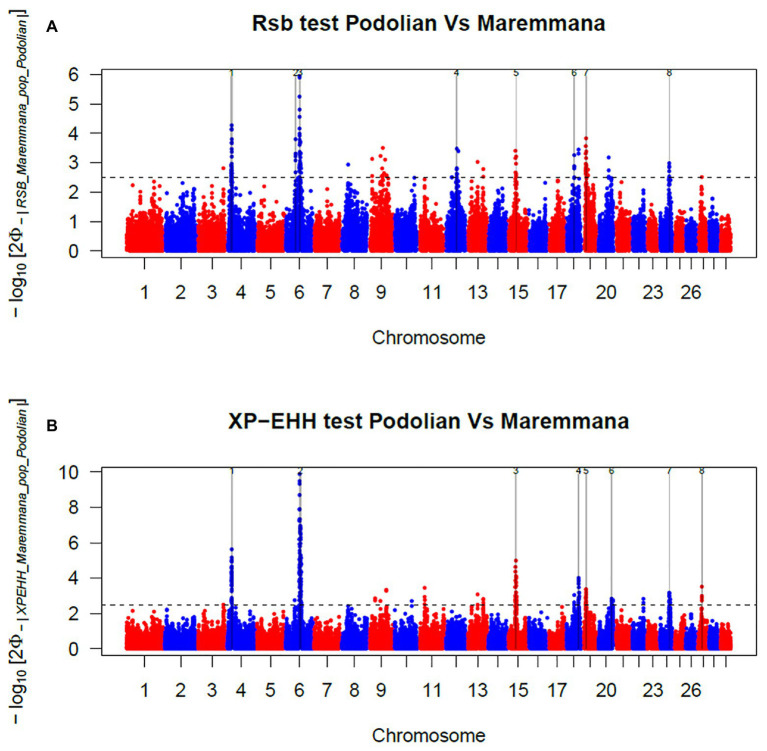
Manhattan plots showing the results of **(A)** *Rsb* and **(B)** cross-population extended haplotype homozygosity (*XP-EHH*) test analyses in the comparison between Maremmana and Italian Podolian-derived breeds. Horizontal dashed lines mark the significance threshold applied to detect the outlier SNPs −log10 (*p*-value) = 2.5.

In total, 396 and 421 outlier SNPs were revealed by *Rsb* and *XP-EHH* tests, respectively (Figures 4A,B), with the two approaches defining eight candidate regions each (Supplementary Tables S2 and S3) and spanning 141 and 219 known protein coding genes. Among these, four genomic regions located on BTA06, BTA15, BTA19, and BTA24 were identified by both approaches (*Rsb* and *XP-EHH*; Table 1). The outlier window on BTA06, spanning 5.54 Mb (at position 58.58–64.12 Mb), showed the strongest signal with 46 and 85% of the SNPs exceeding the significance threshold for the *Rsb* and the *XP-EHH* tests, respectively (Figure 4). The distinctive peak on BTA06 partially overlaps with a run of homozygosity (ROH) island previously detected in Maremmana (Biscarini et al., 2020) and spanning four genes (*KCTD8*, *YIPF7*, *GUF1*, and *GNPDA2*) associated with carcass traits in a composite beef cattle breed (Hay and Roberts, 2018). The *GNPDA2* gene has a potential role in the regulation of body weight, fat, and energy metabolism in chickens (Ouyang et al., 2016) and was associated with adipose tissue accumulation and obesity in humans (Renström et al., 2009). Therefore, this region potentially contributes to the distinctive characteristics of Maremmana related to growth, carcass, and meat traits. The region on BTA06 also showed nine genes (*CHRNA9*, *LIAS*, *N4BP2*, *PDS5A*, *RBM47*, *RHOH*, *SMIM14*, *UBE2K*, and *UGDH*) that overlaps with a ROH island in fast-growing meat commercial hybrid Turkey (Strillacci et al., 2020). Notably, *CHRNA9* gene is also associated with meat quality traits in pigs (Velez-Irizarry et al., 2019). Moreover, the selection signature on BTA06 showed several genes related to mitochondrial functions. For instance, the protein encoded by *LIAS* gene localizes in mitochondrion and is involved in response to oxidative stress (GO:0006979). *GUF1* encodes a GTPase that triggers back-translocation of the elongating ribosome during mitochondrial protein synthesis,^3^ while *UCHL1* is involved in regulation of mitochondrial content and function. Mouse model with skeletal muscle specific knockout of *UCHL1* caused a significant reduction in key proteins that are involved in mitochondrial oxidative phosphorylation in soleus muscles (Gao et al., 2020). This gene is also associated with several other processes such as adult walking behavior (GO:0007628) and eating behavior (GO:0042755). This distinctiveness between Maremmana and the other Podolian-derived Italian breeds is believed to be due to differences in mitochondria-associated gene pathways. Indeed, meat quality of beef is likely to be impacted by breed differences in postmortem mitochondrial respiratory activity within the muscle tissue (Ramos et al., 2020). Furthermore, previous studies have suggested a link between muscle mitochondrial function and feed efficiency in several species, such as chickens (Ojano-Dirain et al., 2005), beef cattle (Fernandez et al., 2020), and pigs (Carmelo and Kadarmideen, 2020). Overall, several of our candidate genes affect multiple, apparently unrelated, phenotypes (pleiotropic effect), which represents one of the difficulties in identifying phenotypic targets of selection.

**Table 1:** Overlapped genomic regions identified in at least two approaches in Maremmana cattle breed.

BTA	Start (Mb)	End (Mb)	Approaches	Genes
4	19.31	22.10	*iHS*, *Rsb* and *XP-EHH*	*THSD7A*, *LOC101902990*, *TMEM106B*, *LOC112446322*, *LOC101906705*, *LOC104971960*, *SCIN*, *LOC100297402*, *ARL4A*, *LOC112446323*, *LOC781728*, *LOC112446324*, *LOC112446494*, *ETV1*
6	58.58	64.12	*Rsb* and *XP-EHH*	*LIAS*, *UGDH*, *SMIM14*, *UBE2K*, *LOC112447195*, *PDS5A*, *TRNAG-CCC*, *LOC112447155*, *N4BP2*, *RHOH*, *CHRNA9*, *LOC112447071*, *RBM47*, *LOC101901948*, *LOC104968867*, *NSUN7*, *APBB2*, *LOC613534*, *UCHL1*, *LIMCH1*, *LOC112447073*, *PHOX2B*, *TMEM33*, *LOC784473*, *SLC30A9*, *LOC782858*, *BEND4*, *LOC101903036*, *LOC107132567*, *LOC112447075*, *SHISA3*, *ATP8A1*, *LOC527955*, *GRXCR1*, *LOC112447156*, *LOC112447157*, *LOC101906152*, *KCTD8*, *YIPF7*, *GUF1*, *GNPDA2*, *LOC104968873*, *LOC112447181*, *LOC112447015*
12	46.31	47.12	*iHS* and *Rsb*	*DACH1*
15	33.01	34.80	*Rsb* and *XP-EHH*	*LOC107131357*, *UBASH3B*, *LOC107133160*, *CRTAM*, *JHY*, *LOC107133161*, *BSX*, *LOC107133162*, *HSPA8*, *LOC112441683*, *LOC112441682*, *CLMP*, *GRAMD1B*, *MIR2313*, *SCN3B*, *ZNF202*, *LOC112441623*, *LOC100847729*, *SAAL1*, *TPH1*, *SERGEF*, *KCNC1*, *MYOD1*
19	14.02	17.36	*Rsb* and *XP-EHH*	*HNF1B*, *LOC101906072*, *LOC104969035*, *LOC101906153*, *HEATR6*, *LOC100847618*, *LOC515676*, *LOC525415*, *LOC100462699*, *LOC100848100*, *WFDC18*, *LOC100296618*, *LOC100847724*, *CCL4*, *LOC107131498*, *CCL3*, *LOC616364*, *LOC100297044*, *LOC112442607*, *LOC504773*, *LOC508666*, *CCL14*, *CCL16*, *CCL5*, *HEATR9*, *TAF15*, *MMP28*, *C19H17orf50*, *GAS2L2*, *RASL10B*, *AP2B1*, *PEX12*, *LOC112442809*, *SLFN14*, *LOC112442608*, *SLFN11*, *LOC101907813*, *LOC100848263*, *LOC112442609*, *LOC112442610*, *LOC112442826*, *LOC112442590*, *LOC112442786*, *UNC45B*, *NLE1*, *FNDC8*, *RAD51D*, *MIR2331*, *RFFL*, *LIG3*, *LOC104969067*, *ZNF830*, *CCT6B*, *LOC112442567*, *LOC112442798*, *TMEM132E*, *LOC789304*, *CCL1*, *CCL8*, *CCL11*, *CCL2*, *LOC112442773*, *ASIC2*, *LOC104969069*, *LOC112442874*, *SPACA3*, *TMEM98*, *MYO1D*
24	39.70	41.54	*Rsb* and *XP-EHH*	*ARHGAP28*, *LAMA1*, *LOC101904673*, *LRRC30*, *LOC781276*, *PTPRM*, *MIR22850-4*, *RAB12*, *MTCL1*, *LOC107131785*, *LOC100849069*, *NDUFV2*, *ANKRD12*

### Relevant Selection Signatures of Distinctiveness in Maremmana Cattle Compared to Podolian-Derived Italian Breeds

A common constraint of selection signature detection methods is the identification of false signals that do not reflect selection (Fijarczyk and Babik, 2015); rather they arise from heterogeneous recombination rates along the genome or owing to several demographic processes such as migration, expansions, and bottlenecks (Vitti et al., 2013). Using multiple approaches and intersecting signals between at least two of them is recommended to reduce the number of false positives (de Villemereuil et al., 2014; Mastrangelo et al., 2020b). Of particular note is that, unlike previous studies (e.g., Bahbahani et al., 2015), we found a high congruence among the three EHH-based methods. This is particularly true for the *iHS* statistic, where two among the three outlier windows identified by this test, were also detected by the *Rsb* statistic (Table 1). To identify additional reliable candidate genes under differential selection between Maremmana and the other Podolian-derived Italian breeds, we focused on the six regions that were detected by at least two methods (Table 1).

#### Genes Associated With Meat Quality Traits

In addition to the aforementioned genes in the selection signature on BTA04 (*ETV1*, *SCIN*, and *THSD7A*), we identified other candidate genes involved in several BP related to muscle compartments, and many of them were previously shown to be associated with meat quality traits. For instance, *MYOD1* on BTA15 (also known as the myogenic differentiation one gene), plays a key role in skeletal muscle cell differentiation (GO:0035914). Polymorphisms within this gene are associated with carcass and meat quality traits in beef cattle (Du et al., 2013). Furthermore, this gene overlapped with a selection region for intramuscular fat and backfat thickness in pig (Kim et al., 2015; Li et al., 2016). Likewise, the *LIMCH1* gene on BTA06 encodes an actin stress fibers-associated protein that activates non-muscle myosin IIA (Lin et al., 2017) and was shown to be under selection in Brahman cattle (Chen et al., 2020). Likewise, two other candidate genes on BTA19, *GAS2L2*, and *unc-45 myosin chaperone B* (*UNC45B*), were among possible candidate genes for 3 day *postmortem* collagen solubility in three Canadian beef cattle breeds (Lei, 2019). In particular, *GAS2L2* is mainly expressed in skeletal muscle. The protein encoded by this gene appears to cross-link microtubules and microfilaments in muscle (Stroud et al., 2014). *UNC45B* is essential for sarcomeric organization and muscle function in very distinct taxonomic groups spanning from *Caenorhabditis elegans* to humans (Donkervoort et al., 2020) and is among a group of genes that showed statistically significant differences in their expression between beef and dairy cattle (Ciecierska et al., 2020). Therefore, our findings partially reflect the different breeding schemes for meat quality traits between Maremmana and the other Podolian-derived Italian breeds. In fact, a previous study using high throughput mRNA sequencing revealed breed specific variations in gene expression of muscle tissues between Maremmana and Chianina (Bongiorni et al., 2016), including two of our relevant candidate genes on BTA06, *UCHL1*, and *SHISA3*.

#### Genes Associated With Immune Response and Local Adaptation

During hundreds of years, the Maremmana population has been exposed to vectors of several pathogens such as *Ixodes ricinus*, *Rhipicephalus bursa*, *Rhipicephalus turanicus*, and *Rhipicephalus sanguineus* (Toma et al., 2015) carrying hemoparasites such as Babesia and Theileria (Toma et al., 2017). Constant exposure to these parasites is expected to exert a selective pressure on Maremmana immunity-related genes. Accordingly, when we conducted enrichment analysis of the 78 protein coding genes identified in at least two approaches (Table 1), we found significant (Benjamini-corrected *p*-value < 0.05) functional enrichment of several BP and molecular function (MF) terms related to a variety of immune responses (Supplementary Table S4). Lymphocyte chemotaxis (GO:0048247, *n* = 7, Benjamini-corrected *p*-value = 1.32 × 10^−7^), monocyte chemotaxis (GO:0002548, *n* = 7, Benjamini-corrected *p*-value = 4.09 × 10^−7^), cellular response to interferon-gamma (GO:0071346, *n* = 7, Benjamini-corrected *p*-value = 1.09 × 10^−6^) and positive regulation of inflammatory response (GO:0050729, *n* = 7, Benjamini-corrected *p*-value = 2.16 × 10^−6^), were the most enriched BP terms. CCR chemokine receptor binding (GO:0048020, *n* = 7, Benjamini-corrected *p*-value = 3.26 × 10^−8^) and chemokine activity (GO:0008009, *n* = 7, Benjamini-corrected *p*-value = 4.07 × 10^−7^) were the most enriched terms under MF (Supplementary Table S4). Over-representation of GO functions playing a central role in the resistance to pathogens is due to the selection signal observed on the BTA19 harboring several chemokine (C-C motif) ligand genes (*CCL4*, *CCL3*, *CCL14*, *CCL16*, *CCL5*, *CCL1*, *CCL8*, *CCL11*, and *CCL2*). Chemokine ligands are crucially involved in both innate and adaptive responses, and help initiating the humoral immune defense (Shin et al., 2015). Through interaction with their receptors, they play a key role in attracting a diverse set of effector leukocytes to inflammatory sites and in recruiting neutrophils, monocytes/macrophages, dendritic cells (DC), and natural killer (NK) cells (Esche et al., 2005). These findings suggest that chemokine (C-C motif) ligand genes were differentially selected in the compared breeds. It seems that significant differences in cytokine production are behind the marked difference in resistance to challenge with *Theileria annulata* between *Bos taurus* and *Bos indicus* cattle subspecies (Glass et al., 2005). Likewise, by inspecting the relevant candidate regions that overlap between statistical methods, we also noted the presence of many genes with immune-related functions (*CRTAM*, *SLFN11*, *RAB12*, and *AP2B1*). The *CRTAM* gene on BTA15 is involved in adaptive immune response (GO:0002250), in positive regulation of cytokine secretion (GO:0001819) and positively regulates natural killer cell mediated cytotoxicity (GO:0045954). The *SLFN11* gene (on BTA19) belongs to the Schlafen protein family, which plays a prominent role in the induction of immune responses (Geserick et al., 2004) and in the regulation of viral replication (Li et al., 2012), while *RAB12* (on BTA24) is directly regulated in response to immune stimuli (Efergan et al., 2016). The *AP2B1* (on BTA19) encodes a protein, which is a part of the AP2 coat assembly protein complex and links clathrin to receptors in the coated vesicles (Online Mendelian Inheritance in Man, 2003). Importantly, a SNP located in the human ortholog of this gene showed a high evidence for association with malaria susceptibility (Band et al., 2019). This is an intriguing outcome and is fairly compelling when considering a possible crucial role in the adaptation of Maremmana cattle to its malarial environment. Several of tick-borne diseases in the Maremma region (such as theileriosis and babesiosis) are also known to result into hypoxia, increased respiratory and heart rates (Razavi et al., 2015; Bal et al., 2016). Thus, it is not surprising that among our candidates, three genes located on the BTA19 (*HEATR9*, *MMP28*, and *ASIC2*), play key roles in pulmonary diseases. The *HEATR9* gene is highly upregulated in lung cells after influenza or respiratory syncytial virus (RSV) infections in humans (Stairiker et al., 2020). The *MMP28* is expressed by macrophages and regulates their recruitment to the lung, thus playing a role in pulmonary disease (Gharib et al., 2014), while *ASIC2* is expressed in the pulmonary arterial smooth muscle cells (Jernigan et al., 2009) and plays an important role in the regulation of pulmonary vascular reactivity and was shown to be under differential selection between African and Northern European Chickens (Fleming et al., 2017). Furthermore, two other candidate genes were also associated with adaptation to high altitude environmental condition (such as hypoxia) in several species: *SERGEF* on BTA15 (Li et al., 2013; Ma et al., 2019) and *NDUFV2* on BTA24 (Ning et al., 2010). *SERGEF* is also among the 48 differentially expressed genes from two breeds of chicken showing extreme differences in hypoxic adaptation reared in Tibet (Hu et al., 2020), whereas *NDUFV2* has been identified as a candidate gene possibly involved in pulmonary hypertension in Black Angus cattle (Newman et al., 2011). The *NDUFV2* gene has been also detected under selection pressure for lipid metabolism in Yaroslavl cattle, and related with local adaptation to survive under poor nutrition conditions in winter (Zinovieva et al., 2020). Additional genes located in the candidate regions on BTA15 (*HSPA8*, *SCN3B*) and BTA19 (*LAMA1*) point toward other aspects of environmental adaptation of Maremmana: *HSPA8* plays a role in thermoregulatory protective mechanisms in cattle and buffalo under tropical environments (Kumar et al., 2015), and associated with cellular response to heat stress in goats (Mohanarao et al., 2014); *SCN3B* is involved in positive regulation of heart rate (GO:0010460) and sensory perception of pain (GO:0019233). This gene is among the genes differentially expressed in response to hyperthermia induced apoptosis in broiler chicken (Coble et al., 2014). Finally, *LAMA1* has been identified as one of candidate genes implicated for environmental adaptation in indigenous Chinese cattle (Gao et al., 2017).

As expected, these findings support a model of complex integrated genomic circuits involving multiple pathways characterizing response mechanisms to environmental challenges in Maremmana. Yet, it seems unlikely that all the genes cited above are a target of selection. It is likely that only some of these genes may be associated with environmental adaptation in Maremmana, while the others would be indirectly influenced by natural selection through genetic hitchhiking.

#### Genes Associated With Morphological Traits

Between population tests are expected to identify candidate genomic regions harboring genes related to some distinctive morphological primitive characteristics of Maremmana (e.g., horn shape and size, sexual dimorphism). However, our candidate regions harbored few morphology-related genes. *Notchless homolog 1* (*NLE1*; BTA19) is the only candidate gene directly involved in skeletal system morphogenesis (GO:0048705). This gene is essential during organogenesis and in particular during axial development in mice (Beck-Cormier et al., 2014). Several genes associated with other morphological traits in Maremmana likely went undetected by the present study either because of insufficient marker density or poor annotation of some of our candidate regions (e.g., the candidate region on the BTA12). The complete functional annotation of the bovine genome and the use of higher density SNP panels or sequencing data would be particularly relevant to improve our fine mapping results.

## Conclusion

In this study, signal consistency across three EHH-based methods was used to identify relevant genomic regions putatively under differential selection between Maremmana and a group of seven other Podolian-derived Italian breeds. Our results suggest that natural selection has shaped several immunity genes involved in both innate and adaptive response to the pathogens inhabiting Maremmana’s natural habitat. Candidate genes associated with growth and meat traits appear to be targets of differential artificial selection between Maremmana and the other Podolian-derived Italian breeds. Information about the location of these regions can be used to detect variants that may underlie important traits of practical interest for the beef industry, and can help improving the competitiveness of the Maremmana cattle, through the implementation of genetic improvement programs for meat traits. Moreover, the candidate genes that confer a selective advantage for adaptative traits provide valuable information for future functional characterization as a starting point to identify causal genetic variants that control environmental adaptation traits for the genetic improvement of commonly used commercial breeds.

## Data Availability Statement

The Illumina BeadChip 50 k SNP genotype data used and analyzed during the current study for the seven cattle breeds are deposited and available at https://www.animalgenome.org/repository/pub/UPIT2018.0607/. The genotyping data of the Maremmana breed presented in this article are not readily available because the raw datasets are property of the Consiglio per la Ricerca in Agricoltura e l’Analisi dell’Economia Agraria CREA and this information is commercially sensitive.

## Author Contributions

SM conceived, planned, and coordinated the study. SB-J, GS, MB, and SM analyzed the data and performed the statistical analysis. EC, RC, GC, and BP contributed with generation of data. FP and BP acquired the funding. SB-J, EC, FP, and SM contributed to the data interpretation. SB-J, GS, EC, RC, GC, MB, FP, BP, and SM discussed the results and made suggestions and corrections. All authors contributed to the article and approved the submitted version.

### Conflict of Interest

The authors declare that the research was conducted in the absence of any commercial or financial relationships that could be construed as a potential conflict of interest.

The handling editor declared a past collaboration with several of the authors EC, RC, FP, and SM.

## Footnotes

^1^http://www.anabic.it/index1.htm

^2^https://david.ncifcrf.gov/

^3^https://www.genecards.org/cgi-bin/carddisp.pl?gene=GUF1
