# Supplementary material for: Genome-Wide Analysis Reveals Selection Signatures Involved in Meat Traits and Local Adaptation in Semi-Feral Maremmana Cattle
Source: Front Genet. 2021 Apr 28;12:675569. doi: 10.3389/fgene.2021.675569 (PMC8113768; doi:10.3389/fgene.2021.675569)
Supplement: Supplementary file 1 [file Data_Sheet_1.PDF]

Supplementary Material

Genome-wide analysis reveals selection signatures involved in meat traits, immune response and local adaptation in semi-feral Maremmana cattle

Slim Ben-Jemaa[1], Gabriele Senczuk[2], Elena Ciani[3], Roberta Ciampolini[4], Gennaro Catillo[5], Mekki Boussaha[6], Fabio Pilla[2], Baldassare Portolano[7], Salvatore Mastrangelo[7]*

[1]Laboratoire des Productions Animales et Fourragères, Institut National de la Recherche Agronomique de Tunisie, University of Carthage, Tunisia
[2]Dipartimento di Agricoltura, Ambiente e Alimenti, University of Molise, Italy
[3]Dipartimento di Bioscienze, Biotecnologie e Biofarmaceutica, University of Bari "Aldo Moro", Italy
[4]Dipartimento di Scienze Veterinarie, University of Pisa, Italy
[5]Consiglio per la Ricerca in Agricoltura e l'Analisi dell'Economia Agraria (CREA), Centro di Ricerca Zootecnia e Acquacoltura, Italy
[6]INRAE, AgroParisTech, University of Paris Saclay, France
[7]Dipartimento di Scienze Agrarie, Alimentari e Forestali, University of Palermo, Italy

*** Correspondence:**
Salvatore Mastrangelo
salvatore.mastrangelo@unipa.it

Supplementary Figure 1. Neighbor-Joining tree relating all the individuals (Maremmana in red; Podolian-derived Italian breeds in blue). The tree was constructed using allele-sharing distances.

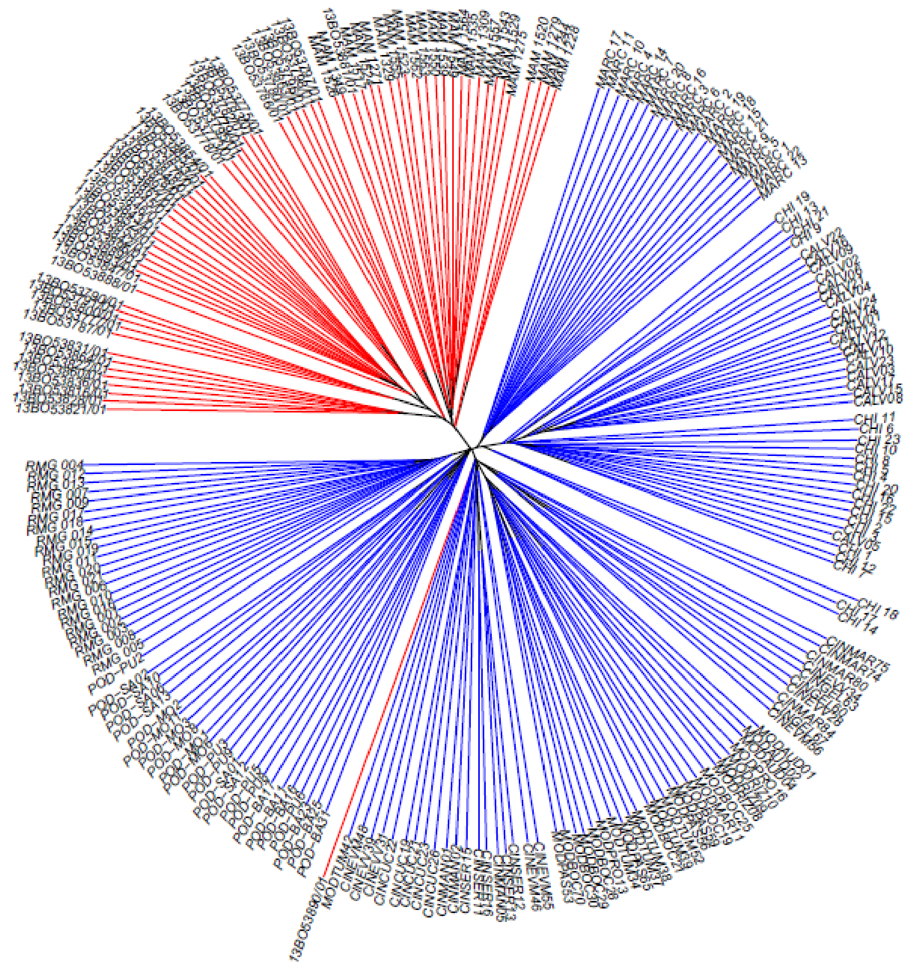

Supplementary Table S1 Genomic regions identified using *iHS* statistic in Maremmana cattle breed.

Region	BTA	Start (bp)	End (bp)	Genes
1	4	18,440,000	22,800,000	LOC781498, LOC1002976616, LOC112446490, LOC783382, NDUFA4, LOC101902990, PHF14, THSD7A, LOC101902990, TMEM106B, VWDE, LOC112446322, LOC101906705, LOC104971960, SCIN, LOC100297402, ARL4A, LOC112446323, LOC781728, LOC112446324, LOC112446494, ETV1, DGKB
2	4	24,620,000	28,600,000	ISPD, SOSTDC1, LRRC72, ANKMY2, LOC112446359, BZW2, TSPAN13, AGR2, AGR3, LOC101907877, AHR, TRNASTOP-UCA, LOC112446489, LOC112446510, LOC112446526, SNX13, LOC112446360, LOC101902492, PRPS1L1, HDAC9, LOC112446506, LOC112446325, LOC112446361, TWIST1, FERD3L, TWISTNB, LOC104971979, TMEM196
3	12	44,980,000	47,120,000	DACH1, TRNAC-ACA

Supplementary Table S2 Genomic regions identified using *Rsb* statistic in the comparisons between Maremmana and Podolian-derived Italian breeds.

Region	BTA	Start (bp)	End (bp)	Genes
1	4	17,570,000	22,100,000	LOC781498, LOC1002976616, LOC112446490, LOC783382, NDUFA4, LOC101902990, PHF14, THSD7A, LOC101902990, TMEM106B, VWDE, LOC112446322, LOC101906705, LOC104971960, SCIN, LOC100297402, ARL4A, LOC112446323, LOC781728, LOC112446324, LOC112446494, ETV1
2	6	42,550,000	44,490,000	LOC112447151, LOC104972733, LOC112447204, PPARGC1A, LOC112447206, DHX15, LOC100138258, LOC104968852, SOD3, CCDC149
3	6	58,580,000	64,120,000	LIAS, UGDH, SMIM14, UBE2K, LOC112447195, PDS5A, TRNAG-CCC, LOC112447155, N4BP2, RHOH, TRNAG-GCC, CHRNA9, LOC112447071, RBM47, LOC101901948, LOC104968867, NSUN7, APBB2, LOC613534, UCHL1, LIMCH1, LOC112447073, TRNAG-CCC, PHOX2B, TMEM33, LOC784473, SLC30A9, LOC782858, BEND4, LOC101903036, LOC107132567, LOC112447075, SHISA3, ATP8A1, LOC527955, GRXCR1, LOC112447156, LOC112447157, LOC101906152, TRNAC-ACA, KCTD8, YIPF7, GUF1, GNPDA2, LOC104968873, TRNAC-ACA, LOC112447181, LOC112447015
4	12	46,310,000	48,300,000	DACH1, TRNAC-ACA, MZT1, BORA, DIS3, PIBF1, KLF5, LOC112449152, LOC112449063
5	15	33,010,000	34,800,000	LOC107131357, UBASH3B, LOC107133160, CRTAM, JHY, LOC107133161, BSX, LOC107133162, HSPA8, LOC112441683, LOC112441682, CLMP, GRAMD1B, MIR2313, SCN3B, ZNF202, LOC112441623, LOC100847729, SAAL1, TPH1, SERGEF, TRNAC-ACA, KCNC1, MYOD1
6	18	32,740,000	34,590,000	CDH11, LOC112442454, CDH5, BEAN1, TK2, LOC104974812, LOC525286, CMTM1, CMTM2, CMTM3, CMTM4, DYNC1LI2, TERB1, NAE1, CA7, PDP2
7	19	14,020,000	18,550,000	HNF1B, LOC101906072, LOC104969035, LOC101906153, HEATR6, LOC100847618, LOC515676, LOC525415, LOC100462699, LOC100848100, WFDC18, LOC100296618, LOC100847724, CCL4, LOC107131498, CCL3, LOC616364, LOC100297044, LOC112442607, LOC504773, LOC508666, CCL14, CCL16, CCL5, HEATR9, TAF15, MMP28, C19H17orf50, GAS2L2, RASL10B, AP2B1, PEX12, LOC112442809, SLFN14, LOC112442608, SLFN11, LOC101907813, LOC100848263, LOC112442609, LOC112442610, LOC112442826, LOC112442590, LOC112442786, UNC45B, NLE1, FNDC8, RAD51D,

				MIR2331, RFFL, LIG3, LOC104969067, ZNF830, CCT6B, LOC112442567, LOC112442798, TMEM132E, LOC789304, CCL1, CCL8, CCL11, CCL2, LOC112442773, ASIC2, LOC104969069, TRNAK-UUU, LOC112442874, SPACA3, TRNAK-CUU, TMEM98, MYO1D, LOC112442877, LOC112442612, LOC101903704, LOC112442611, CDK5R1, PSMD11, ZNF207, C19H17orf75, TRNAQ-UUG, RHBDL3, RHOT1, RNF135, ADAP2, TEFM, ATAD5, CRLF3, LOC101902800, LOC112442816, SUZ12, UTP6, MIR2332, COPRS, LOC112442785, LOC112442862, LOC112442873, LOC112442796, LOC112442613, MIR365-2, MIR2333, MIR193A, TRNAT-CGU, RAB11FIP4
8	24	39,700,000	41,540,000	ARHGAP28, LAMA1, LOC101904673, LRRC30, LOC781276, PTPRM, MIR22850-4, RAB12, MTCL1, LOC107131785, LOC100849069, NDUFV2, ANKRD12

Supplementary Table S3 Genomic regions identified using *XP-EHH* statistic in the comparisons between Maremmana and Podolian-derived Italian breeds.

Region	BTA	Start (bp)	End (bp)	Genes
1	4	19,310,000	22,150,000	THSD7A, LOC101902990, TMEM106B, VWDE, LOC112446322, LOC101906705, LOC104971960, SCIN, LOC100297402, ARL4A, LOC112446323, LOC781728, LOC112446324, LOC112446494, ETV1
2	6	58,380,000	67,220,000	WDR19, LOC112447070, RFC1, KLB, RPL9, LIAS, UGDH, SMIM14, UBE2K, LOC112447195, PDS5A, TRNAG-CCC, LOC112447155, N4BP2, RHOH, TRNAG-GCC, CHRNA9, LOC112447071, RBM47, LOC101901948, LOC104968867, NSUN7, APBB2, LOC613534, UCHL1, LIMCH1, LOC112447073, TRNAG-CCC, PHOX2B, TMEM33, LOC784473, SLC30A9, LOC782858, BEND4, LOC101903036, LOC107132567, LOC112447075, SHISA3, ATP8A1, LOC527955, GRXCR1, LOC112447156, LOC112447157, LOC101906152, TRNAC-ACA, KCTD8, YIPF7, GUF1, GNPDA2, LOC104968873, TRNAC-ACA, LOC112447181, LOC112447015, GABRG1, GABRA2, TRNAW-CCA, LOC100298320, COX7B2, GABRA4, GABRB1, LOC112447219, COMMD8, ATP10D, CORIN, LOC101906472, TRNAC-GCA, LOC104968875, NFXL1, CNGA1, NIPAL1, TXK, TEC, TRNASTOP-UCA, SLAIN2, LOC112447198, SLC10A4, ZAR1, FRYL
3	15	30,160,000	35,270,000	MIR378B, NECTIN1, LOC112441621, TRIM29, OAF, POU2F3, TMEM136, ARHGEF12, GRIK4, MIR2284J, MIR2312, LOC785951, TBCEL, TECTA, LOC104974222, SC5D, LOC112441566, LOC112441622, SORL1, LOC104974223, LOC101903557, LOC100848689, MIR125B-1, MIRLET7A-2, MIR100, LOC107131357, UBASH3B, LOC107133160, CRTAM, JHY, LOC107133161, BSX, LOC107133162, HSPA8, LOC112441683, LOC112441682, CLMP, GRAMD1B, MIR2313, SCN3B, ZNF202, LOC112441623, LOC100847729, SAAL1, TPH1, SERGEF, TRNAC-ACA, KCNC1, MYOD1, OTOG, USH1C, ABCC8, KCNJ11, NCR3LG1, NUCB2, LOC112441567
4	18	50,570,000	52,600,000	DMAC2, ERICH4, LOC614747, LOC100848419, LOC100848940, LOC112442321, LOC112442322, LOC100847120, CEACAM1, LOC112442323, LOC100849237, LIPE, CNFN, MEGF8, TMEM145, PRR19, PAFAH1B3, CIC, ERF, GSK3A, ZNF526, DEDD2, LOC104970208, POU2F2, ZNF574, GRIK5, ATP1A3, RABAC1, LOC101907940, ARHGEF1, CD79A, RPS19, DMRTC2, LYPD4, LOC100336941, LOC112442324, LOC112441502, LOC112442325, LOC522128, LOC112442445, CXCL17, CD177, TEX101, LOC789871, BSP3, BSP5, LOC100299268, BSP1, TRNAA-AGC, LYPD3, PHLDB3, ETHE1, ZNF575, XRCC1,

				LOC506634, LOC616860, IRGQ, ZNF576, ZNF428, SRRM5, LOC107131429, CADM4, PLAUR, LOC512005, LOC526915, IRGC, LOC616722, SMG9, LOC104974883, KCNN4, LYPD5, ZNF283, ZNF404, LOC101903309, ZNF45, ZNF226, ZNF227, ZNF233, ZNF235, ZNF112, ZNF285, LOC112442328, ZNF180, LOC112442327, CEACAM20, LOC112442330, LOC112442423, IGSF23, PVR, CEACAM19, LOC104974891, CEACAM16, LOC107131434, LOC101904248, LOC104974890, BCL3, CBLC, LOC104974889, BCAM, NECTIN2, LOC100141014
5	19	14,020,000	17,360,000	HNF1B, LOC101906072, LOC104969035, LOC101906153, HEATR6, LOC100847618, LOC515676, LOC525415, LOC100462699, LOC100848100, WFDC18, LOC100296618, LOC100847724, CCL4, LOC107131498, CCL3, LOC616364, LOC100297044, LOC112442607, LOC504773, LOC508666, CCL14, CCL16, CCL5, HEATR9, TAF15, MMP28, C19H17orf50, GAS2L2, RASL10B, AP2B1, PEX12, LOC112442809, SLFN14, LOC112442608, SLFN11, LOC101907813, LOC100848263, LOC112442609, LOC112442610, LOC112442826, LOC112442590, LOC112442786, UNC45B, NLE1, FNDC8, RAD51D, MIR2331, RFFL, LIG3, LOC104969067, ZNF830, CCT6B, LOC112442567, LOC112442798, TMEM132E, LOC789304, CCL1, CCL8, CCL11, CCL2, LOC112442773, ASIC2, LOC104969069, TRNAK-UUU, LOC112442874, SPACA3, TRNAK-CUU, TMEM98, MYO1D
6	20	56,810,000	58,590,000	LOC 112442963, MARCH11, TRNAC-ACA, FBXL7, LOC107131578, LOC112443072, ANKH, TRNAG-UCC, OTULIN, OTULINL
7	24	39,490,000	41,650,000	LOC101904513, ARHGAP28, LAMA1, LOC101904673, LRRC30, LOC781276, PTPRM, MIR22850-4, RAB12, MTCL1, LOC107131785, LOC100849069, NDUFV2, ANKRD12, TWSG1, LOC112444203, RALBP1
8	27	16,990,000	18,850,000	LOC101905556, LOC112444655, ZFP42, TRNAG-UCC, TRIML2, TRNAF-AAA, TRIML1, LOC507011, LOC112444625, LOC781928

Supplementary Table S4 Functional annotation clustering results for candidate genes identified by at least two approaches for the Maremmana/ Italian Podolian-derived cattle comparison following DAVID analysis. Significantly enriched functional term clusters (Benjamin-corrected p-value < 0.05) are in bold.

Annotation Cluster 1	Enrichment Score: 4.8798				
Category	Term	Count	PValue	Genes	Benjamini
**GOTERM_MF_DIRECT**	**GO:0048020~CCR chemokine receptor binding**	**7**	**1.25E-10**	**ENSBTAG00000004129, ENSBTAG00000008832, ENSBTAG00000025250, ENSBTAG00000010738, ENSBTAG00000014113, ENSBTAG00000037811, ENSBTAG00000025257**	**1.28E-08**
**GOTERM_BP_DIRECT**	**GO:0048247~lymphocyte chemotaxis**	**7**	**1.69E-10**	**ENSBTAG00000004129, ENSBTAG00000008832, ENSBTAG00000025250, ENSBTAG00000010738, ENSBTAG00000014113, ENSBTAG00000037811, ENSBTAG00000025257**	**5.55E-08**
**GOTERM_BP_DIRECT**	**GO:0002548~monocyte chemotaxis**	**7**	**1.05E-09**	**ENSBTAG00000004129, ENSBTAG00000008832, ENSBTAG00000025250, ENSBTAG00000010738, ENSBTAG00000014113, ENSBTAG00000037811, ENSBTAG00000025257**	**1.73E-07**
**GOTERM_MF_DIRECT**	**GO:0008009~chemokine activity**	**7**	**3.15E-09**	**ENSBTAG00000004129, ENSBTAG00000008832, ENSBTAG00000025250, ENSBTAG00000010738, ENSBTAG00000014113, ENSBTAG00000037811, ENSBTAG00000025257**	**1.60E-07**
**GOTERM_BP_DIRECT**	**GO:0071346~cellular response to interferon-gamma**	**7**	**4.23E-09**	**ENSBTAG00000004129, ENSBTAG00000008832, ENSBTAG00000025250, ENSBTAG00000010738, ENSBTAG00000014113, ENSBTAG00000037811, ENSBTAG00000025257**	**4.64E-07**
**INTERPRO**	**IPR001811:Chemokine interleukin-8-like domain**	**7**	**4.62E-09**	**ENSBTAG00000004129, ENSBTAG00000008832, ENSBTAG00000025250, ENSBTAG00000010738, ENSBTAG00000014113, ENSBTAG00000037811, ENSBTAG00000025257**	**8.60E-07**
**GOTERM_BP_DIRECT**	**GO:0050729~positive regulation of inflammatory response**	**7**	**1.12E-08**	**ENSBTAG00000004129, ENSBTAG00000008832, ENSBTAG00000025250, ENSBTAG00000010738, ENSBTAG00000014113, ENSBTAG00000037811, ENSBTAG00000025257**	**9.21E-07**

**SMART**	**SM00199:SCY**	**7**	**1.20E-08**	**ENSBTAG00000004129, ENSBTAG00000008832, ENSBTAG00000025250, ENSBTAG00000010738, ENSBTAG00000014113, ENSBTAG00000037811, ENSBTAG00000025257**	**6.23E-07**
**GOTERM_BP_DIRECT**	**GO:0070098~chemokine-mediated signaling pathway**	**7**	**1.50E-08**	**ENSBTAG00000004129, ENSBTAG00000008832, ENSBTAG00000025250, ENSBTAG00000010738, ENSBTAG00000014113, ENSBTAG00000037811, ENSBTAG00000025257**	**9.86E-07**
**GOTERM_BP_DIRECT**	**GO:0030593~neutrophil chemotaxis**	**7**	**2.26E-08**	**ENSBTAG00000004129, ENSBTAG00000008832, ENSBTAG00000025250, ENSBTAG00000010738, ENSBTAG00000014113, ENSBTAG00000037811, ENSBTAG00000025257**	**1.06E-06**
**GOTERM_BP_DIRECT**	**GO:0071347~cellular response to interleukin-1**	**7**	**2.26E-08**	**ENSBTAG00000004129, ENSBTAG00000008832, ENSBTAG00000025250, ENSBTAG00000010738, ENSBTAG00000014113, ENSBTAG00000037811, ENSBTAG00000025257**	**1.06E-06**
**GOTERM_BP_DIRECT**	**GO:0071356~cellular response to tumor necrosis factor**	**7**	**8.24E-08**	**ENSBTAG00000004129, ENSBTAG00000008832, ENSBTAG00000025250, ENSBTAG00000010738, ENSBTAG00000014113, ENSBTAG00000037811, ENSBTAG00000025257**	**3.39E-06**
**INTERPRO**	**IPR000827:CC chemokine, conserved site**	**5**	**1.75E-07**	**ENSBTAG00000004129, ENSBTAG00000025250, ENSBTAG00000014113, ENSBTAG00000037811, ENSBTAG00000025257**	**1.62E-05**
**GOTERM_BP_DIRECT**	**GO:0043547~positive regulation of GTPase activity**	**8**	**1.13E-06**	**ENSBTAG00000004129, ENSBTAG00000008832, ENSBTAG00000025250, ENSBTAG00000010738, ENSBTAG00000014113, ENSBTAG00000005340, ENSBTAG00000037811, ENSBTAG00000025257**	**4.14E-05**
**GOTERM_BP_DIRECT**	**GO:0070374~positive regulation of ERK1 and ERK2 cascade**	**7**	**9.84E-06**	**ENSBTAG00000004129, ENSBTAG00000008832, ENSBTAG00000025250, ENSBTAG00000010738, ENSBTAG00000014113, ENSBTAG00000037811, ENSBTAG00000025257**	**3.24E-04**
**UP_KEYWORDS**	**Chemotaxis**	**5**	**1.63E-05**	**ENSBTAG00000004129, ENSBTAG00000025250, ENSBTAG00000014113, ENSBTAG00000037811, ENSBTAG00000025257**	**0.00181**
**GOTERM_BP_DIRECT**	**GO:0006954~inflammatory**	**8**	**1.68E-05**	**ENSBTAG00000004129, ENSBTAG00000008832,**	**5.01E-04**

	**response**			**ENSBTAG00000025250, ENSBTAG00000010738, ENSBTAG00000014113, ENSBTAG00000014520, ENSBTAG00000037811, ENSBTAG00000025257**	
**KEGG_PATHWAY**	**bta04062:Chemokine signaling pathway**	**7**	**2.40E-05**	**ENSBTAG00000004129, ENSBTAG00000008832, ENSBTAG00000025250, ENSBTAG00000010738, ENSBTAG00000014113, ENSBTAG00000037811, ENSBTAG00000025257**	**0.00139**
UP_KEYWORDS	Cytokine	5	9.37E-04	ENSBTAG00000004129, ENSBTAG00000025250, ENSBTAG00000014113, ENSBTAG00000037811, ENSBTAG00000025257	0.05202
UP_KEYWORDS	Inflammatory response	4	0.00144	ENSBTAG00000004129, ENSBTAG00000025250, ENSBTAG00000014113, ENSBTAG00000025257	0.05314
GOTERM_CC_DIRECT	GO:0005615~extracellular space	11	0.00333	ENSBTAG00000018160, ENSBTAG00000000344, ENSBTAG00000004129, ENSBTAG00000008804, ENSBTAG00000008832, ENSBTAG00000025250, ENSBTAG00000010738, ENSBTAG00000009459, ENSBTAG00000014113, ENSBTAG00000037811, ENSBTAG00000025257	0.30342
KEGG_PATHWAY	bta04060:Cytokine-cytokine receptor interaction	5	0.00531	ENSBTAG00000004129, ENSBTAG00000025250, ENSBTAG00000014113, ENSBTAG00000037811, ENSBTAG00000025257	0.15397
GOTERM_BP_DIRECT	GO:0007010~cytoskeleton organization	3	0.03349	ENSBTAG00000004129, ENSBTAG00000025250, ENSBTAG00000037811	0.64819
GOTERM_BP_DIRECT	GO:0008360~regulation of cell shape	3	0.06131	ENSBTAG00000004129, ENSBTAG00000025250, ENSBTAG00000037811	0.84047
GOTERM_BP_DIRECT	GO:0007186~G-protein coupled receptor signaling pathway	7	0.06535	ENSBTAG00000004129, ENSBTAG00000008832, ENSBTAG00000025250, ENSBTAG00000010738, ENSBTAG00000014113, ENSBTAG00000037811, ENSBTAG00000025257	0.85997
UP_KEYWORDS	Disulfide bond	11	0.07338	ENSBTAG00000005193, ENSBTAG00000018160, ENSBTAG00000016768, ENSBTAG00000006086, ENSBTAG00000000344, ENSBTAG00000004129, ENSBTAG00000025250, ENSBTAG00000015252, ENSBTAG00000014113, ENSBTAG00000037811,	1

				ENSBTAG00000025257	
UP_KEYWORDS	Signal	16	0.08011	ENSBTAG00000005193, ENSBTAG00000039504, ENSBTAG00000016768, ENSBTAG00000006086, ENSBTAG00000008832, ENSBTAG00000025250, ENSBTAG00000010738, ENSBTAG00000020046, ENSBTAG00000025257, ENSBTAG00000024015, ENSBTAG00000000344, ENSBTAG00000004129, ENSBTAG00000008804, ENSBTAG00000015252, ENSBTAG00000014113, ENSBTAG00000037811	1
UP_SEQ_FEATURE	disulfide bond	7	0.15686	ENSBTAG00000016768, ENSBTAG00000000344, ENSBTAG00000004129, ENSBTAG00000025250, ENSBTAG00000014113, ENSBTAG00000037811, ENSBTAG00000025257	1
GOTERM_BP_DIRECT	GO:0006955~immune response	3	0.19932	ENSBTAG00000004129, ENSBTAG00000025250, ENSBTAG00000037811	1
UP_KEYWORDS	Secreted	6	0.20971	ENSBTAG00000000344, ENSBTAG00000004129, ENSBTAG00000025250, ENSBTAG00000014113, ENSBTAG00000037811, ENSBTAG00000025257	1
UP_SEQ_FEATURE	signal peptide	7	0.29080	ENSBTAG00000016768, ENSBTAG00000000344, ENSBTAG00000004129, ENSBTAG00000025250, ENSBTAG00000014113, ENSBTAG00000037811, ENSBTAG00000025257	1
Annotation Cluster 2	Enrichment Score: 0.9509				
Category	Term	Count	PValue	Genes	Benjamini
GOTERM_BP_DIRECT	GO:0007264~small GTPase mediated signal transduction	4	0.04070	ENSBTAG00000010356, ENSBTAG00000002869, ENSBTAG00000018518, ENSBTAG00000013451	0.743957491833678
INTERPRO	IPR001806:Small GTPase superfamily	3	0.08219	ENSBTAG00000010356, ENSBTAG00000018518, ENSBTAG00000013451	1
INTERPRO	IPR005225:Small GTP-binding protein domain	3	0.10999	ENSBTAG00000010356, ENSBTAG00000002869, ENSBTAG00000013451	1
GOTERM_MF_DIRECT	GO:0005525~GTP binding	4	0.13225	ENSBTAG00000010356, ENSBTAG00000002869,	1

				ENSBTAG00000018518, ENSBTAG00000013451	
INTERPRO	IPR027417:P-loop containing nucleoside triphosphate hydrolase	6	0.19369	ENSBTAG00000010356, ENSBTAG00000002869, ENSBTAG00000019082, ENSBTAG00000015527, ENSBTAG00000018518, ENSBTAG00000013451	1
UP_KEYWORDS	GTP-binding	3	0.20898	ENSBTAG00000002869, ENSBTAG00000018518, ENSBTAG00000013451	1
Annotation Cluster 3	Enrichment Score: 0.9326				
Category	Term	Count	PValue	Genes	Benjamini
UP_KEYWORDS	Ion channel	4	0.04856	ENSBTAG00000016768, ENSBTAG00000015252, ENSBTAG00000005424, ENSBTAG00000025200	1
UP_KEYWORDS	Ion transport	4	0.16413	ENSBTAG00000016768, ENSBTAG00000015252, ENSBTAG00000005424, ENSBTAG00000025200	1
UP_KEYWORDS	Transport	7	0.19977	ENSBTAG00000016768, ENSBTAG00000020316, ENSBTAG00000004871, ENSBTAG00000015252, ENSBTAG00000005424, ENSBTAG00000019750, ENSBTAG00000025200	1
Annotation Cluster 4	Enrichment Score: 0.7853				
Category	Term	Count	PValue	Genes	Benjamini
INTERPRO	IPR013106:Immunoglobulin V-set	3	0.10046	ENSBTAG00000005193, ENSBTAG00000016768, ENSBTAG00000020046	1
INTERPRO	IPR003599:Immunoglobulin subtype	4	0.12752	ENSBTAG00000005193, ENSBTAG00000024015, ENSBTAG00000016768, ENSBTAG00000020046	1
INTERPRO	IPR013783:Immunoglobulin-like fold	5	0.18668	ENSBTAG00000005193, ENSBTAG00000024015, ENSBTAG00000016768, ENSBTAG00000019092, ENSBTAG00000020046	1
SMART	SM00409:IG	4	0.22184	ENSBTAG00000005193, ENSBTAG00000024015,	1

				ENSBTAG00000016768, ENSBTAG00000020046	
INTERPRO	IPR007110:Immunoglobulin-like domain	4	0.22322	ENSBTAG00000005193, ENSBTAG00000024015, ENSBTAG00000016768, ENSBTAG00000020046	1
Annotation Cluster 5	Enrichment Score: 0.775				
Category	Term	Count	PValue	Genes	Benjamini
UP_KEYWORDS	Nucleotide-binding	9	0.05114	ENSBTAG00000020175, ENSBTAG00000002869, ENSBTAG00000019082, ENSBTAG00000015527, ENSBTAG00000020338, ENSBTAG00000018518, ENSBTAG00000013162, ENSBTAG00000018689, ENSBTAG00000013451	1
UP_KEYWORDS	ATP-binding	6	0.19678	ENSBTAG00000020175, ENSBTAG00000019082, ENSBTAG00000015527, ENSBTAG00000020338, ENSBTAG00000013162, ENSBTAG00000018689	1
GOTERM_MF_DIRECT	GO:0005524~ATP binding	6	0.47000	ENSBTAG00000020175, ENSBTAG00000019082, ENSBTAG00000015527, ENSBTAG00000020338, ENSBTAG00000013162, ENSBTAG00000018689	1
Annotation Cluster 6	Enrichment Score: 0.34749941004790386				
Category	Term	Count	PValue	Genes	Benjamini
UP_KEYWORDS	Iron	3	0.17987	ENSBTAG00000004871, ENSBTAG00000014520, ENSBTAG00000005343	1
UP_KEYWORDS	Metal-binding	7	0.62953	ENSBTAG00000006086, ENSBTAG00000004871, ENSBTAG00000016684, ENSBTAG00000019723, ENSBTAG00000009786, ENSBTAG00000014520, ENSBTAG00000005343	1
KEGG_PATHWAY	bta01100:Metabolic pathways	4	0.80081	ENSBTAG00000004871, ENSBTAG00000014520, ENSBTAG00000014521, ENSBTAG00000005343	1

Annotation Cluster 7	Enrichment Score: 0.2715				
Category	Term	Count	PValue	Genes	Benjamini
UP_KEYWORDS	Ubl conjugation	4	0.30928	ENSBTAG00000002216, ENSBTAG00000020175, ENSBTAG00000013162, ENSBTAG00000015981	1
UP_KEYWORDS	Transcription regulation	3	0.68175	ENSBTAG00000002216, ENSBTAG00000013162, ENSBTAG00000015981	1
UP_KEYWORDS	Transcription	3	0.72685	ENSBTAG00000002216, ENSBTAG00000013162, ENSBTAG00000015981	1
Annotation Cluster 8	Enrichment Score: 0.1339				
Category	Term	Count	PValue	Genes	Benjamini
UP_KEYWORDS	Metal-binding	7	0.62953	ENSBTAG00000006086, ENSBTAG00000004871, ENSBTAG00000016684, ENSBTAG00000019723, ENSBTAG00000009786, ENSBTAG00000014520, ENSBTAG00000005343	1
GOTERM_CC_DIRECT	GO:0005739~mitochondrion	4	0.67693	ENSBTAG00000004871, ENSBTAG00000016684, ENSBTAG00000018689, ENSBTAG00000014520	1
GOTERM_MF_DIRECT	GO:0046872~metal ion binding	3	0.93038	ENSBTAG00000004871, ENSBTAG00000016684, ENSBTAG00000014520	1
Annotation Cluster 9	Enrichment Score: 0.0509				
Category	Term	Count	PValue	Genes	Benjamini
UP_KEYWORDS	Membrane	20	0.71059	ENSBTAG00000007680, ENSBTAG00000005193, ENSBTAG00000016768, ENSBTAG00000014522, ENSBTAG00000020316, ENSBTAG00000004871, ENSBTAG00000002680, ENSBTAG00000005424, ENSBTAG00000020046, ENSBTAG00000025200, ENSBTAG00000005078, ENSBTAG00000024015,	1

				ENSBTAG00000001410, ENSBTAG00000008913, ENSBTAG00000002869, ENSBTAG00000013162, ENSBTAG00000019723, ENSBTAG00000015252, ENSBTAG00000019750, ENSBTAG00000013451	
UP_KEYWORDS	Transmembrane helix	14	0.94118	ENSBTAG00000007680, ENSBTAG00000005193, ENSBTAG00000016768, ENSBTAG00000014522, ENSBTAG00000002680, ENSBTAG00000005424, ENSBTAG00000020046, ENSBTAG00000025200, ENSBTAG00000024015, ENSBTAG00000001410, ENSBTAG00000008913, ENSBTAG00000019723, ENSBTAG00000015252, ENSBTAG00000019750	1
UP_KEYWORDS	Transmembrane	14	0.94323	ENSBTAG00000007680, ENSBTAG00000005193, ENSBTAG00000016768, ENSBTAG00000014522, ENSBTAG00000002680, ENSBTAG00000005424, ENSBTAG00000020046, ENSBTAG00000025200, ENSBTAG00000024015, ENSBTAG00000001410, ENSBTAG00000008913, ENSBTAG00000019723, ENSBTAG00000015252, ENSBTAG00000019750	1
GOTERM_CC_DIRECT	GO:0016021~integral component of membrane	10	0.99131	ENSBTAG00000007680, ENSBTAG00000024015, ENSBTAG00000001410, ENSBTAG00000014522, ENSBTAG00000008913, ENSBTAG00000002680, ENSBTAG00000005424, ENSBTAG00000019750, ENSBTAG00000020046, ENSBTAG00000025200	1
